# Probing Hf_0.5_Zr_0.5_O_2_ Ferroelectricity: Neutron Reflectivity Reveals Critical Interface Effects

**DOI:** 10.1021/acsami.4c18056

**Published:** 2025-02-28

**Authors:** Hsing-Yang Chen, Chi-Lin Mo, Jing-Jong Shyue, Tzu-Yen Huang, Miin-Jang Chen

**Affiliations:** † Department of Materials Science and Engineering, National Taiwan University, Taipei, Taiwan 10617, R.O.C.; ‡ Research Center for Applied Sciences, Academia Sinica, Taipei, Taiwan 11529 R.O.C.; § Neutron Group, National Synchrotron Radiation Research Center, Hsinchu, Taiwan 300, R.O.C; ∥ Graduate Institute of Electronics Engineering, National Taiwan University, Taipei, Taiwan 10617, R.O.C.; ⊥ Graduate School of Advanced Technology, National Taiwan University, Taipei, Taiwan 10617, R.O.C.

**Keywords:** neutron reflection, ferroelectric, Hf_0.5_Zr_0.5_O_2_ (HZO), tungsten oxide, interface

## Abstract

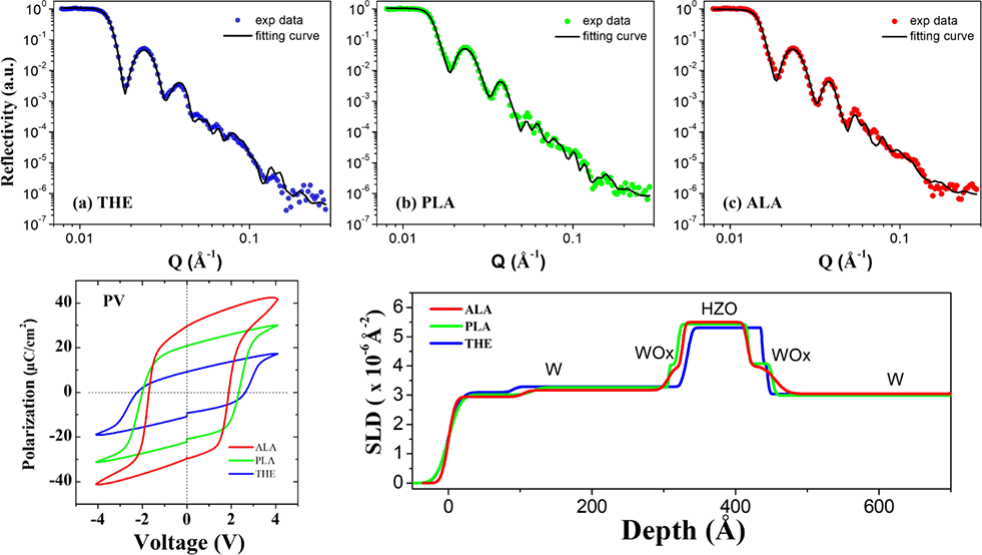

Ferroelectric Hf_0.5_Zr_0.5_O_2_ (HZO) thin films emerge as promising candidates for next-generation memory devices; however, the device performance is strongly correlated to the interfacial structure. In this study, neutron reflectivity was used for the first time to conduct an in-depth analysis of the ferroelectric W/HZO/W devices, leveraging the high sensitivity of neutron scattering length density (SLD) to the buried interfaces. We explored the impact of different atomic layer deposition (ALD) techniques—thermal, plasma, and atomic layer annealing (ALA)—on the structural and ferroelectric characteristics of HZO thin films, with a particular focus on interfacial structures. Analyses using neutron reflectivity, high-resolution transmission electron microscopy, and X-ray photoelectron spectroscopy revealed the critical role of the bottom WOx interfacial layer. The ALA treatment contributes to significant enhancements in structural and ferroelectric properties, including an increase in film density and crystallinity, as well as a maximum neutron SLD due to reduced oxygen vacancies. This work elucidates the crucial role of interface engineering via interfacial layer formation in stabilizing the ferroelectric phase, providing valuable insights for the development of advanced ferroelectric devices.

## Introduction

The discovery of ferroelectricity in Si-doped HfO_2_ thin films by Böscke et al. in 2011 marked a significant milestone that triggered substantial progress in ferroelectric research.^1^ This breakthrough not only unveiled a novel functionality of HfO_2_ but also a paradigm shift, enabling the integration of ferroelectric materials into complementary metal-oxide-semiconductor (CMOS) processes and paving the way for new applications of HfO_2_ in nanoelectronics.^2^ In order to stabilize the metastable ferroelectric orthorhombic phase (o-phase, *Pca*2_1_) in HfO_2_-based thin films, the doping of various elements, such as Al, Gd, La, N, Sr, Y, and Zr, has been extensively investigated in previous studies.^3−5^ Among these dopants, Zr is of particular interest, as it belongs to the same group as Hf in the periodic table and therefore has similar physical and chemical properties. This inherent similarity allows the incorporation of high concentrations of Zr while preserving robust ferroelectric properties.^6^ In addition, the introduction of Zr into HfO_2_ thin films can effectively reduce the crystallization temperature required for the formation of the ferroelectric o-phase.^7^ This low-temperature crystallization capability enables the integration of Hf_0.5_Zr_0.5_O_2_ (HZO) thin films into ferroelectric random-access memory (FeRAM),^8^ ferroelectric field-effect transistors (FeFET),^9^ and ferroelectric tunnel junctions (FTJ)^10^ without compromising compatibility with back-end-of-line processes.^6,11,12^

Recent research has been dedicated to facilitating the formation of the ferroelectric o-phase in HZO thin films. Although the ferroelectric o-phase is not a thermodynamically stable state,^13−16^ this metastable phase can be stabilized through techniques including stress engineering and surface energy modulation.^13,15,17^ These phase stabilization strategies often involve careful control of annealing conditions,^18^ film thickness,^19^ interfacial engineering,^20,21^ and electrode material selection.^22−25^ In particular, the in-plane tensile stress, induced by the mismatch in thermal expansion coefficients between the electrode and the HZO layer during thermal annealing, is a critical factor in stabilizing the desired ferroelectric o-phase.^22,26^ Compared to the commonly used TiN electrode, tungsten (*W*) electrodes have distinct benefits when used in HZO metal-ferroelectric-metal (MFM) devices. The low thermal expansion coefficient of *W* (∼4.5 × 10^–6^ K^–1^) allows for higher in-plane stress within the HZO thin film, thereby favoring the formation of the ferroelectric o-phase and suppressing nonferroelectric phases.^27,28^ Furthermore, studies have demonstrated that HZO MFM devices using *W* electrodes exhibit robust ferroelectricity with high remanent polarization (*P*_r_).^28−31^ However, the deposition process can inadvertently cause oxidation of the underlying *W* electrode, resulting in the formation of the WOx interface. Despite its potential impact on the ferroelectric properties of HZO MFM devices, research efforts specifically investigating the effects of this WOx interfacial layer remain relatively scarce in the existing literature. As the thickness of ferroelectric thin films continues to scale down, interface-related issues become increasingly critical in determining the overall device behavior and functional properties. Consequently, there is a need for comprehensive investigations to elucidate the impact of the WOx interfacial layer on the ferroelectric performance of HZO MFM devices.

In this study, neutron reflectometry (NR) was employed to characterize and analyze the WOx interfacial layer. NR is a nondestructive specular neutron scattering technique that provides depth-resolved information on films and interfaces.^32^ Neutrons, being electrically neutral, have excellent penetration capabilities due to their minimal interaction with electron clouds. Instead, neutrons interact directly with atomic nuclei, with the interaction distance defined as the neutron scattering length. It is notable that the scattering length varies nonmonotonically with atomic number, thereby enabling NR to distinguish between materials with different atomic numbers, even those that are adjacent on the periodic table.^33,34^ In addition, the majority of materials exhibit relatively low neutron absorption cross-sections, with the exception of a few elements, including boron, cadmium, and lithium. Due to the weak interactions between neutrons and most materials, NR is an advantageous technique for studying buried interfaces.^34^ In summary, the unique benefits of NR arise from the irregular relationship between neutron scattering length and atomic number, coupled with the generally low neutron absorption by most materials. These properties allow for straightforward penetration of neutrons through thick solid materials to probe complex interfaces and multilayer structures that may be challenging to analyze with alternative techniques.

On the other hand, HZO thin films are mainly deposited via atomic layer deposition (ALD) due to their advantages such as precise thickness control, conformal coverage, and large-area uniformity. In conventional ALD processes, deposition temperatures are typically kept below 300 °C to maintain the essential self-limiting growth, but such temperatures are often insufficient to achieve optimal ferroelectric properties in HZO. Increasing the deposition temperature could be detrimental to thin film quality as a result of precursor decomposition and failure of the self-limiting mechanism at excessively high temperatures.^35^ In previous studies, the novel concept of atomic layer annealing (ALA) was introduced, which enabled the realization of high-quality crystalline thin films at low deposition temperatures. The ALA process incorporates additional plasma treatments using inert gases, specifically argon (Ar) or helium (He), in each ALD cycle. The use of inert gas plasma mitigates undesired chemical reactions and enhances surface reactions and atomic migration, thereby significantly improving film crystallinity.^36−41^ For example, the ALA treatment implemented in each ALD cycle can facilitate low-temperature epitaxial growth of AlN and GaN, even at temperatures as low as 300 °C.^38,42^ Further investigations have demonstrated that the ALA method can increase film density and improve the dielectric constant,^41^ highlighting the critical role of ALA in the ALD process.

This study investigates the ferroelectric properties of HZO thin films prepared by thermal (THE), plasma (PLA), and ALA ALD techniques, using *W* as the electrode material. For the first time, NR measurements were used to elucidate the effects of different ALD processes on the HZO/W interface, exploiting the significant contrast in neutron scattering length densities (SLD) between WO_3_ (∼4.1 × 10^–6^ Å^–2^) and *W* (∼3.0 × 10^–6^ Å^–2^) to provide insights into the interface structure. The study reveals a distinct WOx interfacial layer between the electrode and the HZO layer, prepared by both the PLA and ALA ALD processes. X-ray photoelectron spectroscopy (XPS) analysis indicates that the HZO thin films prepared by the PLA and ALA methods have significantly lower oxygen vacancy densities, suggesting that the WOx layer serves as a source of oxygen. The NR data fitting revealed that the HZO film prepared with the ALA process has the maximum SLD, indicating a superior film density. The higher SLD correlates well with a lower number of oxygen vacancies, leading to enhanced ferroelectric properties in the ALA-prepared HZO thin film.

## Experimental Methods

**Figure 1:**
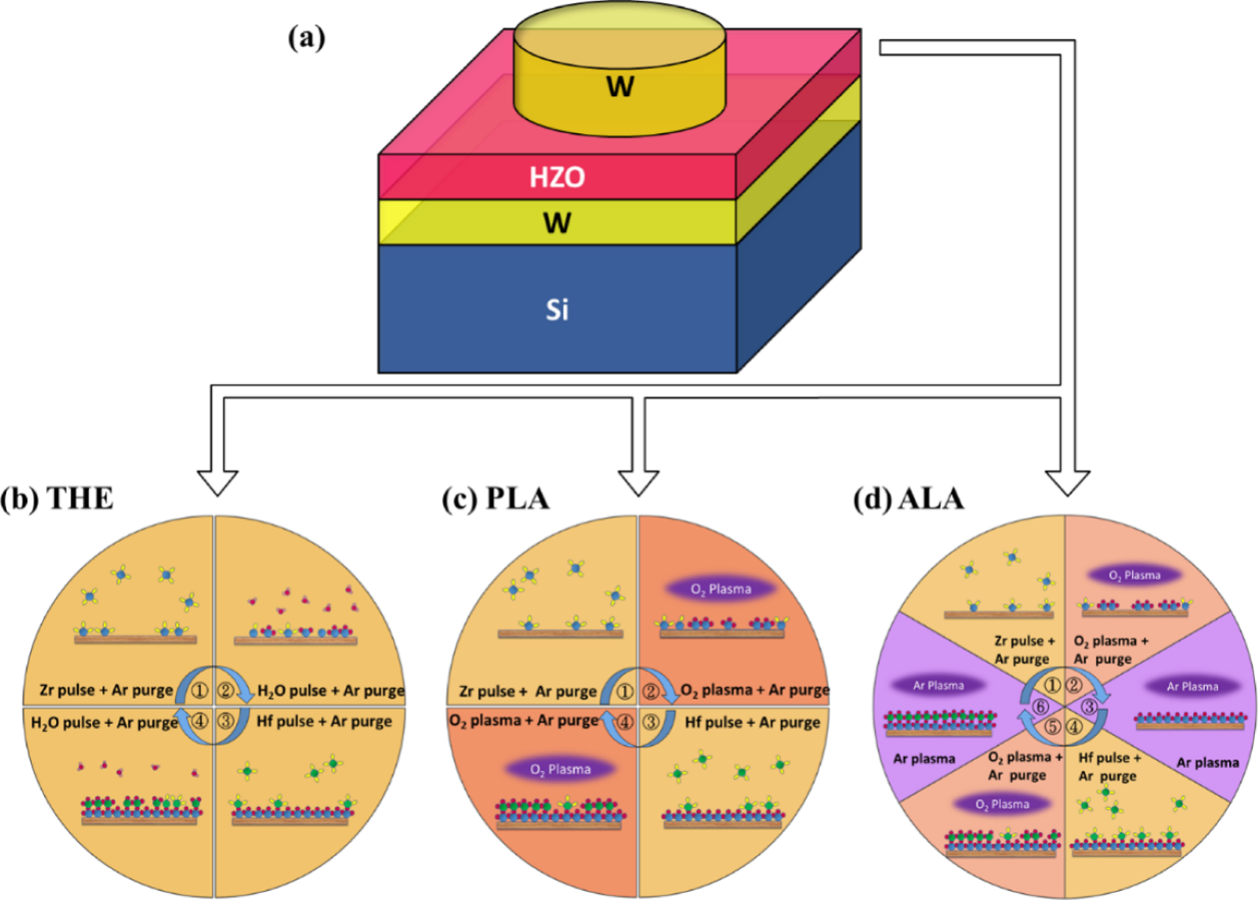
(a) Schematic diagrams of the MFM device structure. (b, c, d) The ALD cycles of the THE, PLA, and ALA processes for the deposition of HZO in the (b) THE-, (b) PLA-, and (d) ALA-samples. Note that the Ar plasma treatments are carried out after the deposition of each HfO_2_ and ZrO_2_ monolayer in the ALA process.

Figure 1a shows the schematic diagrams of the MFM devices. The *W* bottom electrode was deposited by sputtering on an *n*-type Si substrate, as the boron-doped *p*-type Si wafer is unsuitable for NR characterization due to the high neutron absorption cross-section of boron. The ALD process was then carried out at 300 °C using the Fiji system (Cambridge NanoTech) to deposit an HZO layer with a thickness of ∼9–10 nm on the *W* bottom electrode. Tetrakis(dimethylamido)zirconium and tetrakis(dimethylamido)hafnium were used as precursors for Zr and Hf, respectively. The oxygen sources were selected according to the specific process employed, with water vapor and oxygen plasma being used in the THE and PLA ALD processes, respectively. For the ALA process, a layer-by-layer, *in situ* argon (Ar) plasma treatment with 50-W radio frequency power was incorporated after the deposition of each HfO_2_ and ZrO_2_ monolayer within the conventional plasma ALD cycle. A complete overview of the ALD cycles used to prepare HZO thin films by the THE, PLA, and ALA methods is illustrated in Figure 1b–d, where the specimens are referred to as THE-, PLA-, and ALA- samples, respectively. Square *W* top electrodes, each with an area of 75 × 75 μm^2^, were prepared via sputtering and then patterned using conventional photolithography and liftoff processes. Afterward, annealing was performed at 450 °C in an N_2_ atmosphere using a rapid thermal annealing system.

High-resolution transmission electron microscopy (HRTEM) images were obtained using a JEOL 2010F microscope equipped with a 200-kV field-emission gun to examine the MFM structures. X-ray photoelectron spectroscopy (XPS) was used to explore oxygen vacancies in the HZO thin films. This measurement was conducted using a PHI VersaProbe scanning microprobe with a monochromatic Al Kα X-ray source (1486.6 eV) and an energy resolution of 0.2 eV. All XPS spectra were calibrated by aligning the C 1s peak to 284.8 eV. The XPS spectra were then analyzed using XPSPEAK41 software with Shirley background subtraction and Gaussian/Lorentzian mixed functions for peak fitting. The MFM devices were electrically characterized by using a semiconductor parameter analyzer (Keithley 4200-SCS). The polarization-voltage (*P–V*) and current–voltage *(I–V*) loops were measured using triangular bipolar voltage pulses at a frequency of 2 kHz. The capacitance–voltage (*C–V*) curves were probed at 10 kHz. Quantitative assessments of the switching field distributions were performed using first-order reversal curve (*FORC*) analysis, in which the *FORC* minor loops were generated by first saturating the devices and then cycling between the saturation and various reversal voltages. The positive-up-negative-down (*PUND*) technique, which utilizes two consecutive positive and negative electrical pulses, was employed to directly measure the polarization hysteresis loops.

The NR measurements were conducted on the Spatz time-of-flight neutron reflectometer at the Australian Nuclear Science and Technology Organization (ANSTO) with a 20-MW OPAL research reactor.^43^ The selected chopper pairings were set to a rotation speed of 25 Hz, which provided a wavelength resolution of about 5%. The NR data were collected at two incident angles of 0.70° and 3.50° to cover the range of momentum transfer *q* (0.008 ≤ *q* ≤ 0.28 Å^–1^). The *q* value is given by *q* = 4π sin(θ)/λ, where θ is the angle of incidence and λ is the wavelength. An illuminated footprint 18 mm long and 30 mm wide was used. Data reduction was performed using Jupyter Notebook with the *refnx* package.^44^ The procedure included correcting the detector efficiency, converting the time-of-flight data to the *q* value, and stitching the data sets from the two incident angles to provide a complete reflectivity profile with scaling the critical edge equal to unity. The NR data were analyzed using the *refnx* software.^44^

## Results and Discussion

**Figure 2:**
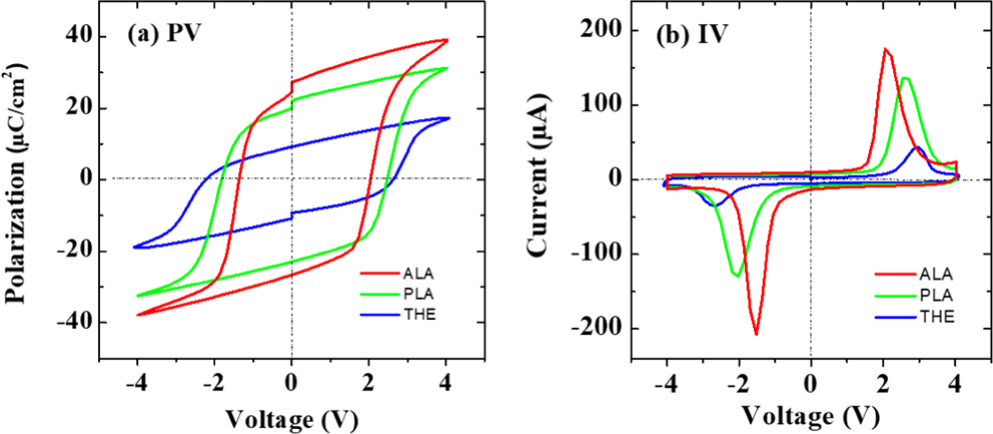
(a) *P–V* and (b) *I–V* loops of the THE-, PLA-, and ALA-MFM devices. The ALA-sample demonstrated the highest remanent polarization and switching current among the tested specimens.

**Figure 3:**
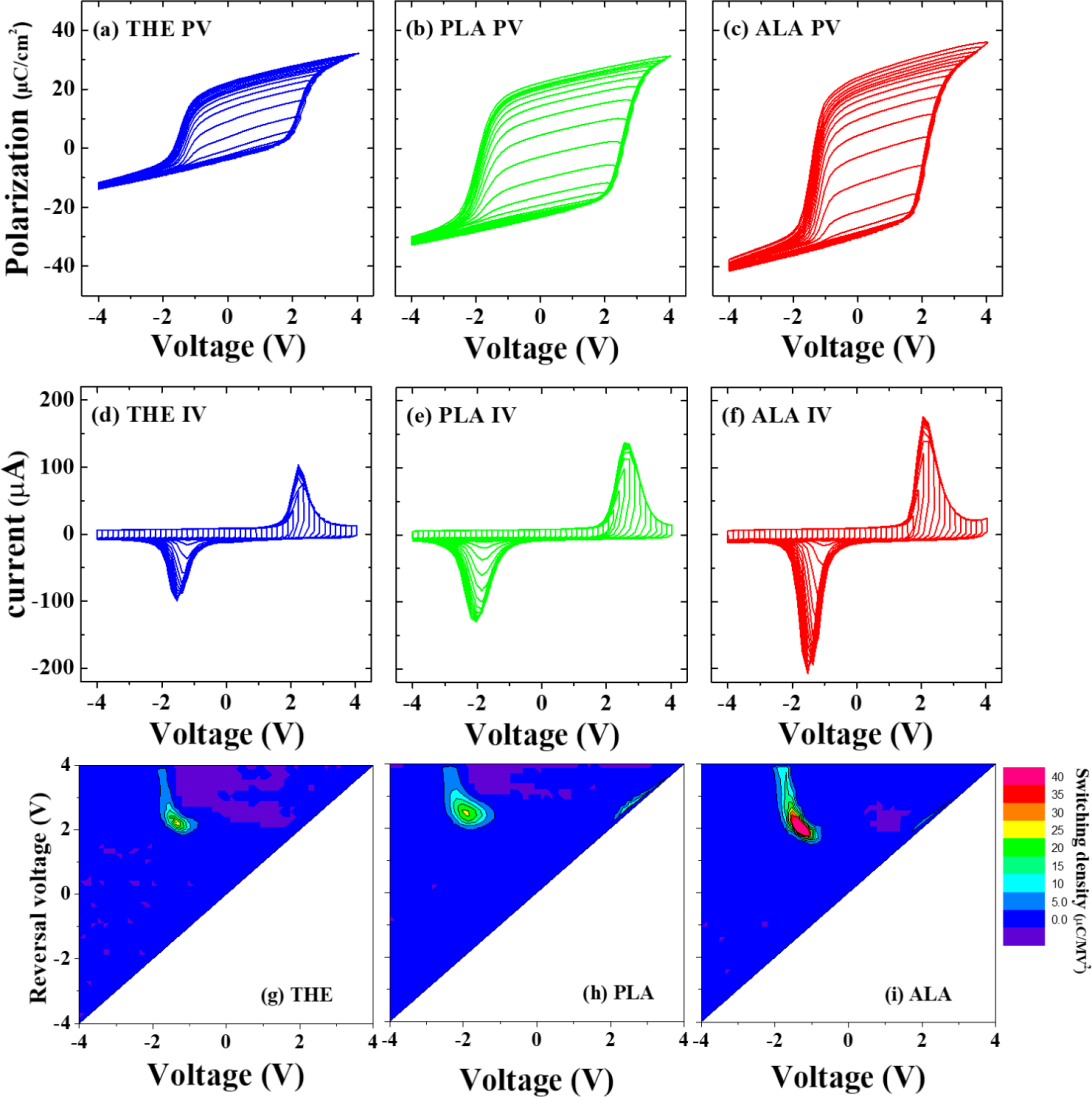
Sweeps of the *P–V* hysteresis loops for (a) THE-, (b) PLA-, and (c) ALA-devices, which were conducted with different reversal voltages to generate the *FORC* plots, along with their corresponding *I–V* curves in (d), (e), and (f), respectively. The *FORC* distribution diagrams of the (g) THE-, (h) PLA-, and (i) ALA-MFM devices. The *FORC* measurements were initiated at a saturation voltage of −4 V, swept incrementally toward the reversal voltage, and terminated at a positive saturation at +4 V, with a constant voltage sweep rate of 5 × 10^4^ V/s. The color scale in (g), (h), and (i) illustrates the switching probability density (ρ).

The *P–V* hysteresis loops and *I–V* curves of the THE-, PLA-, and ALA-MFM devices in their pristine state are shown in Figure 2. The most significant remanent polarization (*P*_r_) and switching current were observed in the ALA-sample, indicating superior ferroelectric characteristics of the HZO layer prepared using the ALA process. Then, the *FORC* measurements were conducted on the THE-, PLA-, and ALA-samples to analyze the distribution of polarization switching in the HZO layers.^45,46^Figure 3a–f shows a series of *P–V* and *I–V* curves, each corresponding to a specific reversal voltage. As revealed in Figure 3g–i, the *FORC* distributions of the three MFM devices in the pristine state display a single, concentrated peak of polarization switching. This pattern suggests a high degree of coherence in the ferroelectric switching of the HZO layers. In addition, the *P–V* hysteresis loops shown in Figure 2 remain nearly unchanged after being subjected to 10^3^ electrical cycles, demonstrating that the W/HZO/W devices exhibit wake-up-free behavior. This indicates that electrical cycling is not required to activate the ferroelectric properties of the HZO thin films prepared by the three different ALD processes. The endurance characteristics of the THE-, PLA-, and ALA-MFM devices are shown in Figure S1 in Section S1.

**Figure 4:**
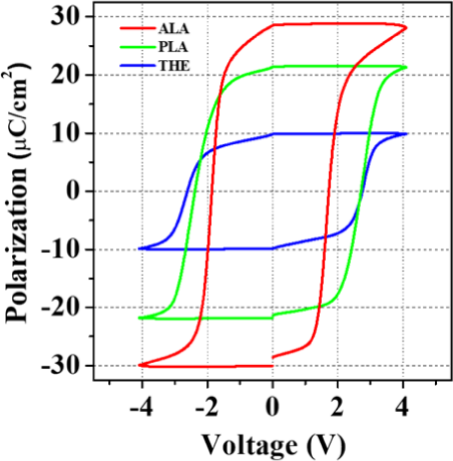
*PUND* tests of the THE-, PLA-, and ALA-MFM devices, which were performed using a maximum applied voltage of 4 V and a pulse frequency of 2 kHz. It can be seen that the ALA treatment leads to the most remarkable ferroelectricity.

Figure 4 shows the *P–V* curves of the pristine-state THE-, PLA-, and ALA-MFM devices, which were obtained through *PUND* measurement. The *PUND* method can effectively eliminate nonferroelectric switching currents, including leakage and capacitive contributions, thereby enabling the precise extraction of 2*P*_r_.^47^ The *PUND* results indicate that the 2*P*_r_ values of the ALA-, PLA-, and THE- samples are 57.9, 43.0, and 19.7 μC/cm^2^, respectively. A maximum 2*P*_r_ value was observed in the ALA-device, indicating that the HZO thin film prepared with the ALA treatment may contain a higher amount of ferroelectric o-phase, thereby exhibiting a more pronounced ferroelectric response.

**Figure 5:**
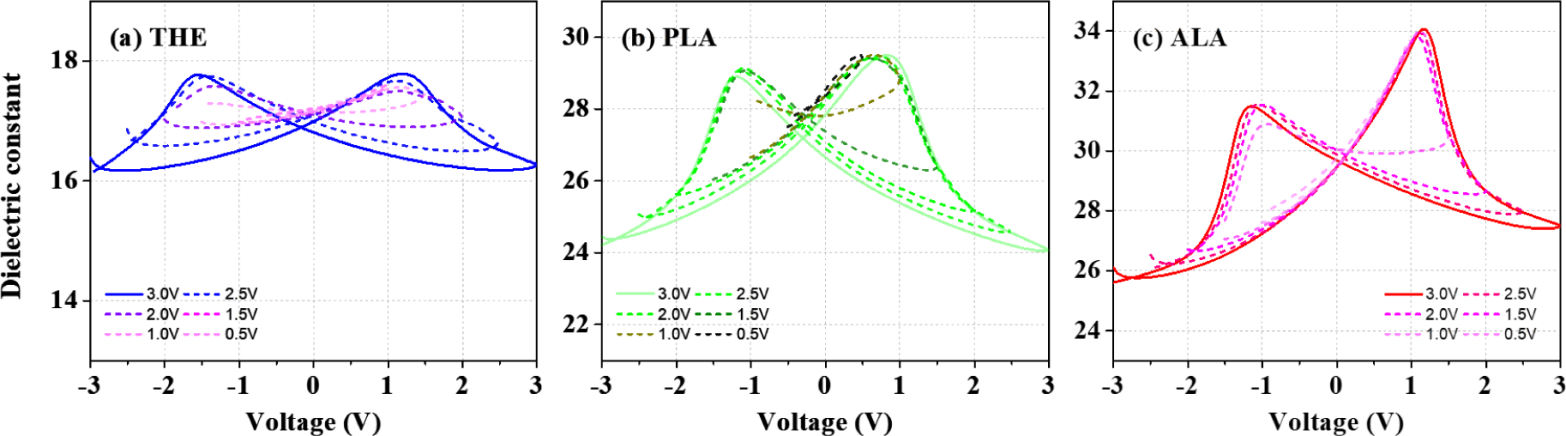
*ε*_r_–*V* curves of the (a) THE-, (b) PLA-, and (c) ALA-MFM devices. Each plot shows the curves measured over a number of different voltage ranges, as indicated in the legend.

Figure 5 presents the dielectric constant versus voltage (*ε*_r_*–V*) curves of the THE-, PLA-, and ALA- MFM devices, where the *ε*_*r*_ values were derived from the *C–V* measurement. The PLA- and ALA-samples exhibit significant ferroelectric characteristics with distinctive butterfly-like shapes. The dielectric constants of the HZO layers in the ALA-, PLA-, and THE-devices at −3 V, as indicated in the *ε*_r_*–V* curves, are approximately 26, 24, and 16, respectively. It is noteworthy that the *ε*_r_ values of the PLA- and ALA-samples are close to the reported dielectric constant of the ferroelectric o-phase, which is typically in the range of 25–30.^12,48^ This suggests that the ferroelectric o-phase is predominant in the PLA- and ALA-samples. In contrast, the lower *ε*_r_ value and the absence of a butterfly-like curve of the THE-sample may be ascribed to the relatively low crystallinity within the HZO layer.^49^

**Figure 6:**
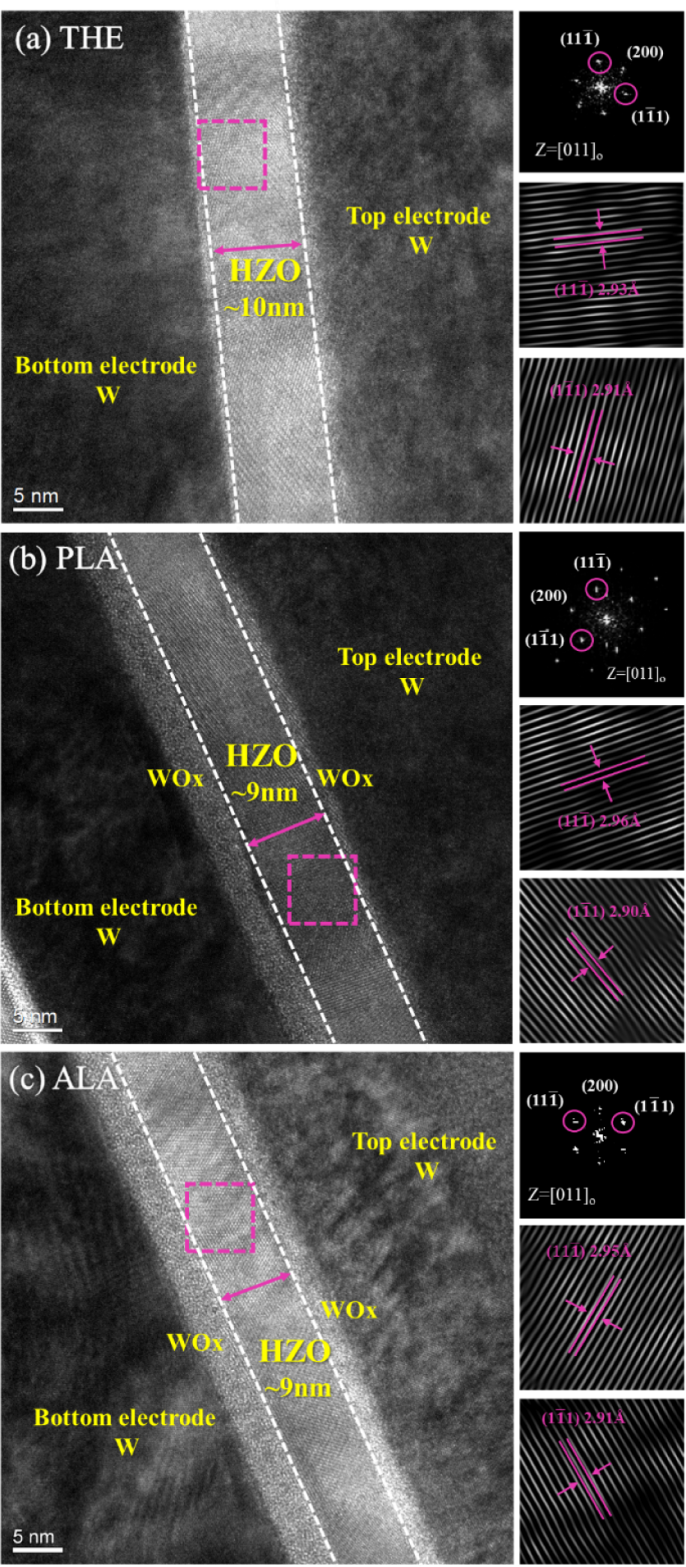
HRTEM micrographs (taken at 100k× magnification) of the (a) THE-, (b) PLA-, and (c) ALA-samples. Insets display the FFT diffractograms of the HZO crystalline regions (magenta rectangles), with indexed diffraction spots revealing the crystal structure. Two distinct diffraction spots were selected for inverse FFT processing, which yields *d*-spacing and lattice fringes corresponding to planes approximately parallel and perpendicular to the HZO layer.

The HRTEM images of the MFM structures in the THE-, PLA-, and ALA-samples are shown in Figure 6. The amorphous regions at the interface between the HZO and *W* electrodes in these images can be identified as the WOx layer. In the THE-sample, the WOx interfacial layer adjacent to the bottom electrode appears less pronounced (Figure 6a). In contrast, a distinct WOx layer can be observed at the interface between HZO and the *W* bottom electrode in both the PLA- and ALA-samples (Figure 6b,c). This difference may be attributed to the exposure of O_2_ plasma during the PLA and ALA ALD processes, which facilitates the oxidation of the *W* bottom electrode and results in the formation of the WOx interfacial layer.

The relative degree of crystallinity can be distinguished by examining the lattice fringes of the HZO layer in the HRTEM images. The ALA-sample exhibits the highest degree of crystallinity, followed by the PLA-sample, and then the THE-sample, which is consistent with the ferroelectric properties observed in the electrical measurements. Fast Fourier transform (FFT) analysis of the HRTEM images, conducted by using Digital Micrograph software, identifies the o-phase diffraction patterns along the [011] zone axis in the HZO layers of all three samples. Inverse FFT operations performed on the diffraction spots near the in-plane and out-of-plane directions give the corresponding *d*-spacing values. This analysis indicates that the *d*-spacing near the in-plane direction is larger than that near the out-of-plane direction for all samples. This observation suggests that the *W* electrode imposes a tensile stress on the HZO layer along the in-plane direction, thereby stabilizing the o-phase and inducing ferroelectricity in HZO.^27^Figure S2 in Section S2 shows the grazing incidence X-ray diffraction patterns, providing nonlocal crystallographic information on the THE-, PLA-, and ALA-samples.

**Figure 7:**
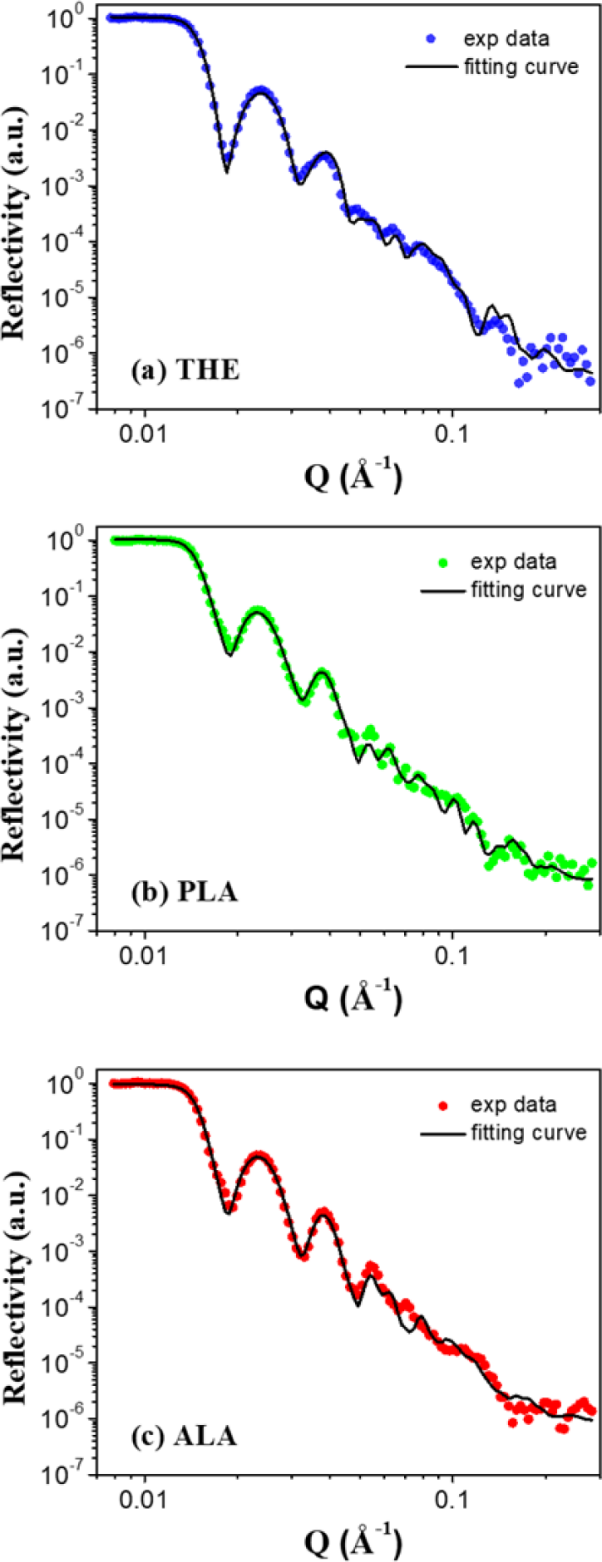
NR profiles and fitting curves of the (a) THE-, (b) PLA-, and (c) ALA-samples.

**Figure 8:**
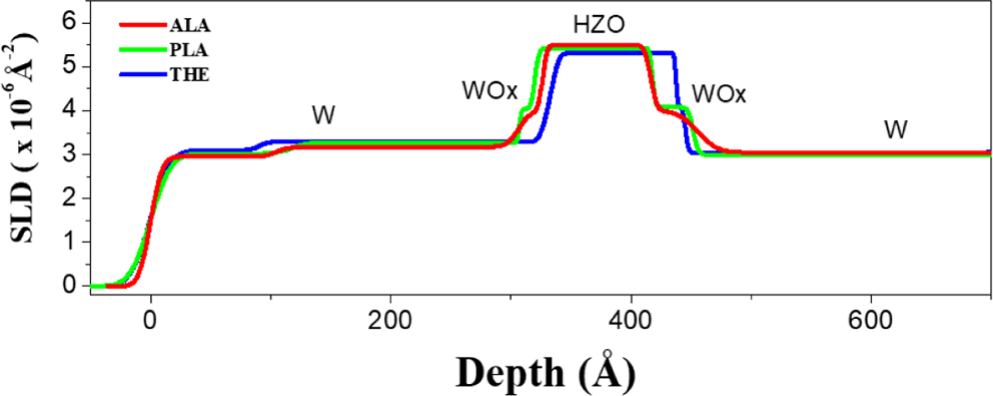
SLD profiles of the THE-, PLA-, and ALA-samples, which were extracted from the experimental NR data. It can be seen that the WOx interfacial layer at the bottom interface of the ALA-sample has a greater thickness compared to that of the THE- and PLA-samples. In addition, the SLD of the HZO layer reaches its highest value in the ALA-sample.

Due to the excellent penetration capability of neutrons, NR is a powerful tool that can provide valuable insight into the W/HZO/W device structure. Figure 7 shows the NR profiles of THE-, PLA-, and ALA-samples. The analysis and fitting of the NR data were performed using the *refnx* software, with appropriate constraints on the SLD values and initial estimates for the layer thickness according to the HRTEM images. The NR analysis yields quantitative information on the thickness and neutron SLD of each layer. The resulting fitting curves demonstrate excellent agreement with the experimental NR data, as revealed in Figure 7. Figure 8 presents the SLD depth profiles of the MFM structures, which were derived from the curve-fitting analysis of the NR data. In the THE-sample, an abrupt interface is observed between the HZO and W layers, indicative of a well-defined and chemically distinct heterointerface. The thicknesses of the WOx interfacial layers obtained by curve fitting are all below the nanoscale in the THE-sample. This observation suggests minimal interdiffusion and compositional inhomogeneity at the HZO/W boundary in the THE-sample. In contrast, in the PLA- and ALA- samples that have been subjected to plasma treatments, significant compositional gradients are present in the WOx interfacial layer, revealing the formation of diffuse interfacial transition regions. Notably, the SLD profile indicates that the WOx interfacial layers in the ALA-sample are thicker than those in the PLA-sample. Specifically, the thicknesses of the WOx layers adjacent to the top and bottom electrodes are 20 and 39 Å, respectively, for the ALA-sample, while they are 13 and 34 Å for the PLA-sample. The observed differences in SLD may result from the higher surface temperature induced by the Ar plasma exposure during the ALA treatment. The SLD values of the HZO films are 5.5 × 10^–6^ Å^–2^, 5.4 × 10^–6^ Å^–2^, and 5.3 × 10^–6^ Å^–2^ for the ALA-, PLA-, and THE-samples, respectively. These variations in SLD arise from the differences in the film density and stoichiometric composition among the samples. The maximum SLD observed in the ALA-sample can be primarily attributed to the following factors: (1) the additional Ar plasma exposure during the ALA treatment increases film densification and improves crystallinity,^36,38,39,41^ and (2) the presence of a distinct WOx interfacial layer at the HZO/W bottom interface. This interfacial layer contributes oxygen atoms to the HZO layer during the annealing process,^50−52^ effectively reducing oxygen vacancies and consequently increasing the SLD of the HZO layer in the ALA-sample.

**Figure 9:**
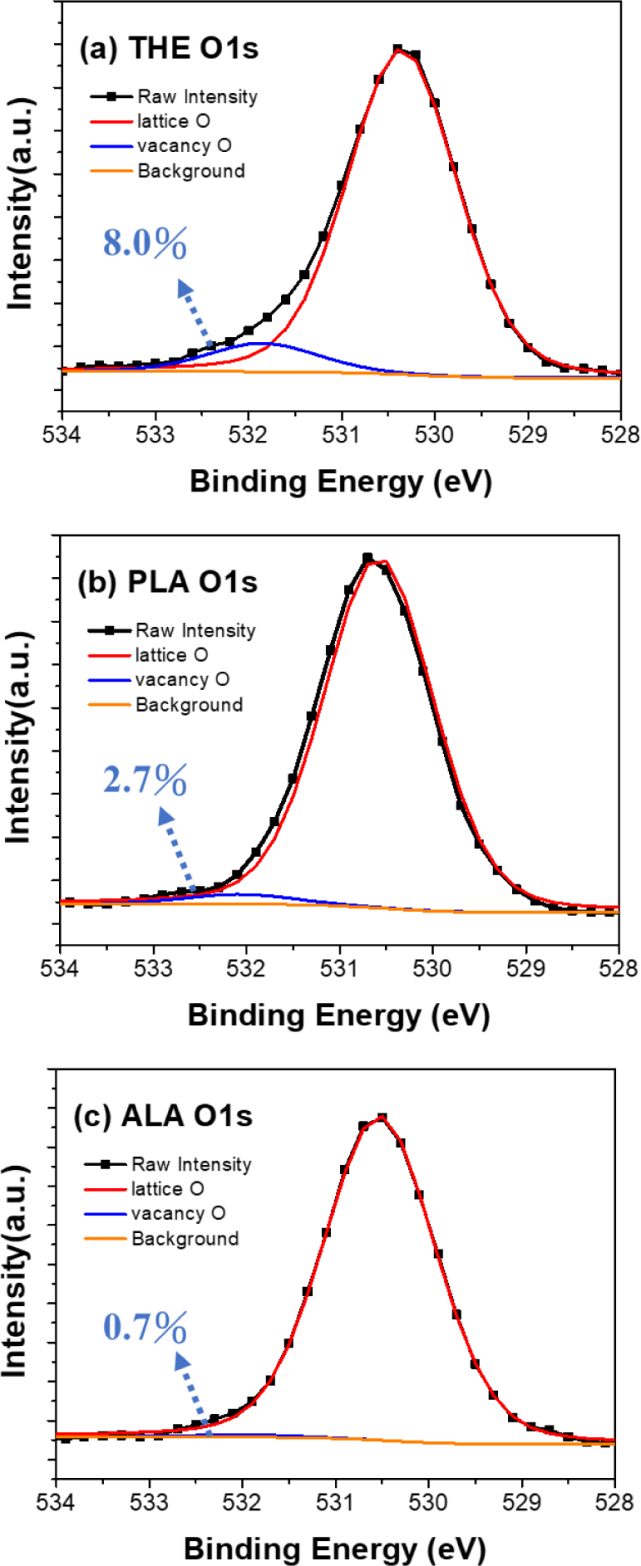
O 1s XPS spectra of the (a) THE-, (b) PLA-, (c) ALA-HZO thin films, along with the deconvolution into two peaks centered at 530.5 ± 0.1 eV and 532.0 ± 0.1 eV, respectively. A low amount of oxygen vacancies of only ∼0.7% was observed in the ALA-sample.

The discussion of oxygen vacancies in the previous paragraph can be supported by XPS analysis. Figure 9 shows the deconvoluted O 1s spectra of the THE-, PLA-, and ALA-samples with two distinct peaks centered at 530.5 ± 0.1 eV and 532.0 ± 0.1 eV, which are correlated with lattice oxygen and oxygen vacancies in the HZO layer, respectively.^53,54^ The quantification of oxygen vacancies can be achieved by calculating the ratio of the integrated area associated with oxygen vacancies to the total area of the O 1s spectra. This analysis reveals significant differences in the proportion of oxygen vacancies among the samples: 8.0%, 2.7%, and 0.7% for the THE-, PLA-, and ALA- samples, respectively. From a thermodynamic perspective, the Gibbs free energy of formation for WO_3_ (−8.7 eV)^55^ is less negative than that of HfO_2_ (−12.31 eV)^56^ and ZrO_2_ (−11.71 eV).^56^ As a result, WOx is more prone to supplying oxygen atoms to HZO during rapid thermal annealing.^57^ This thermodynamic property is further supported by the SLD profile analysis shown in Figure 8. In the ALD processes involving PLA or ALA treatments, the formation of the WOx interfacial layer could serve as an oxygen reservoir. During the annealing process, oxygen atoms diffuse from the WOx layer into the HZO film, effectively reducing oxygen vacancies and thereby contributing to a higher SLD of HZO.^58,59^ On the other hand, the wake-up-free characteristics observed in the devices, as shown in Figure 2, can be ascribed to the low concentration of oxygen vacancies as reported in previous studies.^18,60,61^ The presence of oxygen vacancies is primarily responsible for the wake-up effect in ferroelectric HZO devices.^62^ Extensive literature has indicated that the concentration of oxygen vacancies plays a critical role in stabilizing crystalline phases in HZO.^58,63,64^ An appropriate concentration of oxygen vacancies is beneficial for the formation of the ferroelectric o-phase, whereas an excessive concentration (>18%) favors the tetragonal phase.^59,63^ Density Functional Theory (DFT) calculations suggest that the ferroelectric o-phase is effectively stabilized at a low oxygen vacancy concentration of about 0.93%.^51^ Experimentally, the dose of oxygen source used in the ALD process can be adjusted to modulate the oxygen content in the HZO films.^58,63^ It is shown that optimal ferroelectric properties in HZO thin films are achieved with an oxygen vacancy concentration of approximately 0.6%,^63^ which is close to the content of oxygen vacancies in the ALA-sample. The Hf 4f and Zr 3d XPS analyses of the HZO thin films in the THE-, PLA-, and ALA- samples are presented in Figure S3 in Section S3, demonstrating the qualitative agreement in the oxygen vacancy content with the O 1s XPS analysis. In summary, the observed oxygen vacancy concentration in the ALA-sample falls within the optimal range for stabilizing the o-phase of HZO, which is in good agreement with both theoretical predictions and previous experimental results. This highlights the importance of carefully controlling the oxygen vacancy concentration to achieve significant ferroelectric performance of HZO thin films.

## Conclusion

This study utilized NR, a technique characterized by its exceptional sensitivity to buried interfaces, to probe the interfacial modifications in the W/HZO/W structure, comprising W, WOx, and HZO layers. THE, PLA, and ALA ALD techniques were employed to synthesize ferroelectric HZO thin films in order to elucidate their impact on the interfacial and ferroelectric properties. The NR analysis revealed the presence of distinct bottom WOx interfacial layers in the PLA- and ALA-samples. The WOx interfacial layer acts as an oxygen source, which is favorable for reducing the concentration of oxygen vacancies in HZO during the annealing process. This reduction is crucial for ferroelectric performance by stabilizing the desired ferroelectric o-phase. In contrast, the THE-sample, which was prepared by a thermal ALD process without a plasma-induced interfacial layer, exhibited inferior ferroelectricity. The absence of a significant WOx interfacial layer results in a higher concentration of oxygen vacancies in HZO, which, in turn, degrades the ferroelectric properties. Notably, the ALA treatment can significantly improve both the density and crystallinity of the HZO layer, and the presence of the WOx interfacial layer effectively suppresses oxygen vacancies in HZO films. As a result, a maximum SLD and the most pronounced ferroelectricity were observed in the ALA sample, indicating a substantial improvement in both the structural and electrical properties achieved by the ALA method. In summary, this study has demonstrated that NR is an effective method for probing complicated interface structures in depth. The results provide valuable insights into ferroelectric materials and highlight the critical role of interface engineering in the development of advanced ferroelectric devices.

## Supporting Information Available

Additional studies provide detailed information on the structural, chemical, and ferroelectric properties of the HZO thin films, thereby supporting the main conclusions of this research (PDF)
