# Supplementary material for: Probing Hf0.5Zr0.5O2 Ferroelectricity: Neutron Reflectivity Reveals Critical Interface Effects
Source: ACS Appl Mater Interfaces. 2025 Feb 28;17(10):16102–10. doi: 10.1021/acsami.4c18056 (PMC11912186; doi:10.1021/acsami.4c18056)
Supplement: Supplementary file 1 — am4c18056_si_001.pdf [file am4c18056_si_001.pdf]

Supporting Information

Probing $Hf_{0.5}Zr_{0.5}O_2$ ferroelectricity: neutron reflectivity reveals critical interface effects

Hsing-Yang Chen[1], Chi-Lin Mo[1], Jing-Jong Shyue[2], Tzu-Yen Huang[3,*],

and Miin-Jang Chen[1,4,5,*]

[1] Department of Materials Science and Engineering, National Taiwan University, Taipei 10617, Taiwan, R.O.C.

[2] Research Center for Applied Sciences, Academia Sinica, Taipei, Taiwan, R.O.C.

[3] Neutron Group, National Synchrotron Radiation Research Center, Hsinchu, Taiwan, R.O.C

[4] Graduate Institute of Electronics Engineering, National Taiwan University, Taipei 10617, Taiwan, R.O.C.

[5] Graduate School of Advanced Technology, National Taiwan University, Taipei 10617, Taiwan, R.O.C.

*Author to whom any correspondence should be addressed.

E-mail: mjchen@ntu.edu.tw and huang.ty@nsrrc.org.tw

Section 1: The endurance characteristics of the THE-, PLA-, and ALA- MFM devices

To provide a clear and more intuitive representation of the endurance behavior, the endurance characteristics of the THE-, PLA-, and ALA- MFM devices were evaluated using bipolar triangular voltage waveforms at a frequency of 2 kHz. Figure S1(a) shows the evolution of the remanent polarization (P_r) under voltage cycling stress of ±3 V amplitude. It can be seen that the P_r values of all three devices do not significantly increase with the endurance cycles, indicating that the HZO thin films in this study are almost free of the wake-up operation. In particular, the ALA- MFM device reveals exceptionally stable P_r from the initial endurance cycles, demonstrating its wake-up-free behavior.

The endurance characteristics in Figure S1(a) show that the P_r values remain stable to ~10^2, 10^5, and 10^4 endurance cycles for the THE-, PLA-, and ALA- MFM devices, respectively. The reduced endurance performance observed in the ALA-sample can be attributed to its high P_r. Previous studies have reported that high ferroelectric polarization is typically associated with a significant degradation in endurance.[S1,S2] This correlation can be understood through the continuity of the displacement field (D) at the interface, as shown in Equation (1) below:

$$D = \varepsilon_0 \varepsilon_F E_F + P_S = \varepsilon_0 \varepsilon_{IL} E_{IL} \qquad (1)$$

where ε_0 is the vacuum permittivity, ε_F and ε_{IL} are the dielectric constants of the ferroelectric and interfacial layers, E_F and E_{IL} are the respective electric fields, and P_S denotes the ferroelectric spontaneous polarization. The equation shows that a higher P_S increases the E_{IL}, thereby accelerating the degradation of endurance properties.[S3] This behavior manifests an inverse correlation between the maximum achievable polarization switching cycles and the remanent polarization in ferroelectric materials. Furthermore, ferroelectric materials with high P_r require stronger electric fields for polarization switching, resulting in increased operating stress and thus accelerated fatigue.

It should be noted from Figure 2 in the manuscript that the coercive voltage of the ALA-MFM device is only about 2V. As a result, additional fatigue endurance tests were conducted on the ALA-device at reduced operating voltages, as shown in Figure S1(b). When subjected to lower applied voltages of 2.5 V and 2.0 V, the device demonstrates improved endurance, achieving ~10^5 and 10^6 switching cycles, respectively. This improvement in endurance performance at lower voltages is due to the reduced polarization switching magnitude.

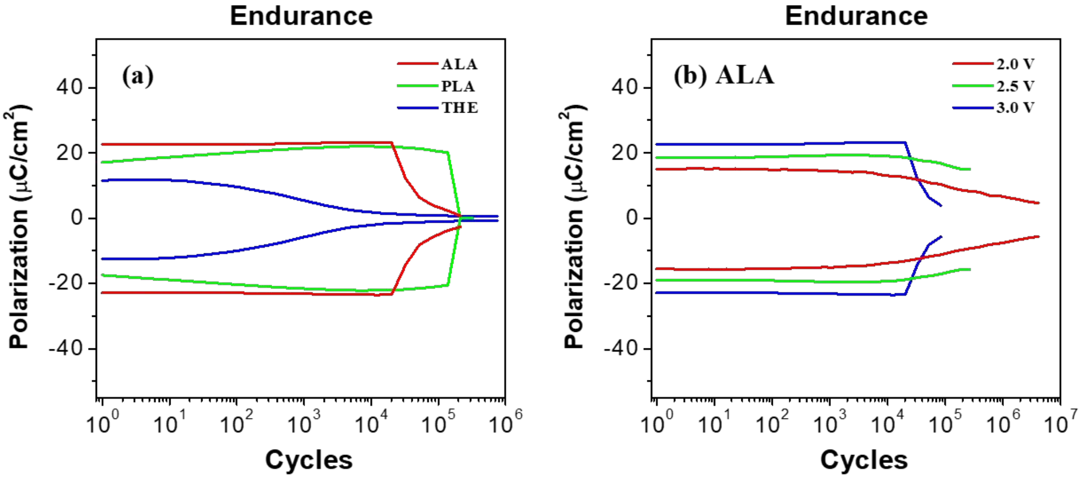

Figure S1. The evolution of polarization as a function of the number of switching cycles (fatigue endurance characteristics) for (a) the THE-, PLA-, and ALA- MFM devices, and (b) the ALA-device under different applied voltages (2.0, 2.5, and 3.0 V).

Section 2: The GIXRD patterns of the THE-, PLA-, and ALA- samples

The grazing incidence X-ray diffraction (GIXRD) patterns were measured at an incident angle of 0.5° to analyze the crystallographic structure of the THE-, PLA-, and ALA- samples. As shown in Figure S2, the patterns reveal dominant body-centered cubic tungsten XRD peaks near 2θ~40° (JCPDS #04-0806), along with several characteristic diffraction peaks corresponding to HZO phases. These include the monoclinic m-($\bar{1}11$) peak at 2θ ~ 28.6°, the mixed orthorhombic/tetragonal (o-/t-) peaks at 30.4° (o-(111)/t-(011)), 35.4° (o-(200)/t-(002)), and 44° (o-(112)/t-(012)).[S4] It can be seen that the ALA-sample exhibits the most significant peak intensities of the mixed o-/t- phase, whereas the THE-sample shows minimal intensity. This crystallographic observation is consistent with the ferroelectric hysteresis measurement in Figure 2, suggesting that these diffraction peaks originate primarily from the o-phase. Furthermore, the absence of the wake-up effect, as shown by the endurance test in Figure S2, further supports the dominance of the ferroelectric o-phase in the film. This is because the wake-up effect in HZO is typically associated with the t-to-o phase transition, which was not observed here.[S5] Although weak m-($\bar{1}11$) peaks were detected in both the ALA- and PLA- samples, their much lower intensities relative to the dominant o-phase peaks suggest a negligible influence on the overall material properties. The GIXRD analysis reveals the dominant diffraction peaks from the o-

phase in the ALA-sample, which correlates well with its most pronounced ferroelectric performance as demonstrated by the *P-V*, *FORC*, and *PUND* characteristics.

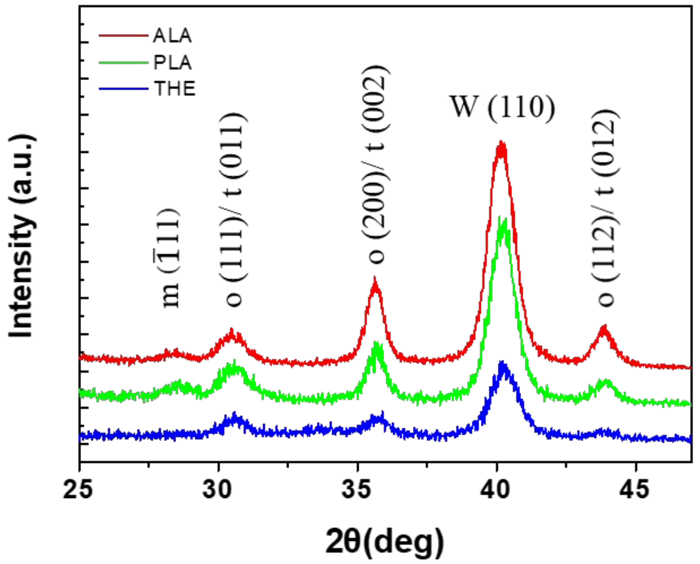

Figure S2. GIXRD patterns of the THE-, PLA-, and ALA- samples.

Section 3: Hf 4f and Zr 3d XPS analyses of the HZO thin films in the THE-, PLA-, and ALA- samples

XPS analysis was conducted to examine the chemical states of Hf and Zr within the HZO layer, as shown in Figure S3. The spectra were deconvoluted using characteristic spin-orbit splitting energies of 1.68 eV and 2.43 eV for Hf 4f and Zr 3d, respectively, with intensity ratios of 3:4 (Hf $4f_{5/2}$:$4f_{7/2}$) and 2:3 (Zr $3d_{3/2}$:$3d_{5/2}$). The Hf 4f spectra (Figure S3 (a)-(c)) display the primary Hf $4f_{5/2}$ peak at 17.5 eV corresponding to stoichiometric HfO_2 and a secondary peak shifted by ~1.4 eV toward lower binding energies, associated with the presence of oxygen-deficient HfO_{2-x}.[18,24] Similarly, the Zr 3d spectra (Figure S3 (d)-(f)) reveal the main Zr $3d_{3/2}$ peak at 182.3 eV for stoichiometric ZrO_2, along with a ~1.5 eV shift attributed to oxygen-deficient ZrO_{2-x}.[S6]

Quantitative analysis of the oxygen vacancy contents, determined from the ratio of the suboxide peak area to the total spectral peak area, reveals distinct differences between the deposition methods. The Hf-related oxygen vacancy contents are 7.9%, 5.7%, and 3.3% for the THE-, PLA-, and ALA- samples, respectively. Similarly, the Zr-related oxygen vacancy contents are 12.4%, 9.7%, and 5.5% for the THE-, PLA-, and ALA- samples. These results are in qualitative agreement with the O1s XPS analysis, confirming that the ALA treatment contributes to a significant suppression of the oxygen vacancy concentration in the HZO thin film.

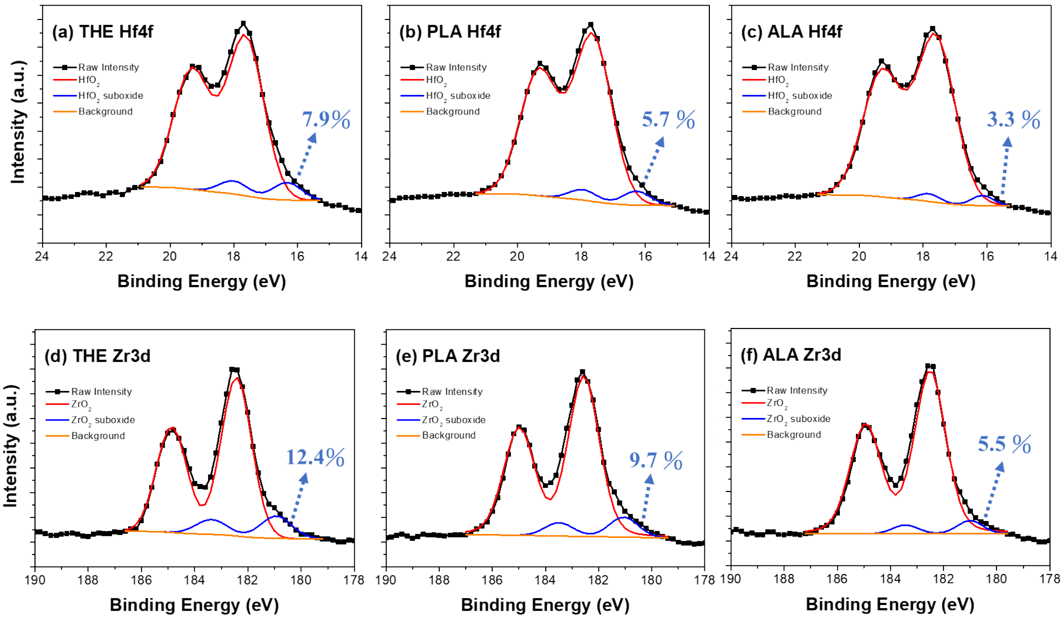

Figure S3. (a-c) Hf 4f and (d-f) Zr 3d XPS spectra of HZO thin films in the THE-, PLA-, and ALA- samples. Peak deconvolution reveals the presence of HfO_2/HfO_{2-x} and ZrO_2/ZrO_{2-x} components, indicating a mixture of fully oxidized and oxygen-deficient oxide phases. Notably, the ALA-sample has significantly lower oxygen vacancy contents compared to the THE- and PLA- samples.

References

(S1) Park, M. H.; Lee, Y. H.; Mikolajick, T.; Schroeder, U.; Hwang, C. S. Review and Perspective on Ferroelectric HfO_2-Based Thin Films for Memory Applications. *MRS Communications* **2018**, *8* (3), 795-808. https://doi.org/10.1557/mrc.2018.175.

(S2) Chernikova, A. G.; Kozodaev, M. G.; Negrov, D. V.; Korostylev, E. V.; Park, M. H.; Schroeder, U.; Hwang, C. S.; Markeev, A. M. Improved Ferroelectric Switching Endurance of La-Doped $Hf_{0.5}Zr_{0.5}O_2$ Thin Films. *ACS Appl Mater Interfaces*. **2018**, *10* (3), 2701-2708. https://doi.org/10.1021/acsami.7b15110.

(S3) Mulaosmanovic, H.; Breyer, E. T.; Dünkel, S.; Beyer, S.; Mikolajick, T.; Slesazeck, S. Ferroelectric Field-Effect Transistors Based on HfO_2: A Review. *Nanotechnology* **2021**, *32* (50), 502002. https://doi.org/10.1088/1361-6528/ac189f.

(S4) Kashir, A.; Farahani, M. G.; Lančok, J.; Hwang, H.; Kamba, S. A Grease for Domain Walls Motion in HfO_2-Based Ferroelectrics. *Nanotechnology* **2022**, *33* (15), 155703. https://doi.org/10.1088/1361-6528/ac4679.

(S5) Saini, B.; Huang, F.; Choi, Y. Y.; Yu, Z.; Thampy, V.; Baniecki, J. D.; Tsai, W.; McIntyre, P. C. Field-Induced Ferroelectric Phase Evolution During Polarization "Wake-Up" in $Hf_{0.5}Zr_{0.5}O_2$ Thin Film Capacitors. *Adv Electron Mater* **2023**, *9* (6), 2300016. https://doi.org/10.1002/aelm.202300016.

(S6) Barreca, D.; Battiston, G. A.; Gerbasi, R.; Tondello, E.; Zanella, P. Zirconium Dioxide Thin Films Characterized by XPS. *Surface Science Spectra* **2000**, *7* (4), 303-309. https://doi.org/10.1116/1.1375573.
